# Supplementary material for: Biomedical researchers’ perspectives on the reproducibility of research
Source: PLoS Biol. 2024 Nov 5;22(11):e3002870. doi: 10.1371/journal.pbio.3002870 (PMC11537370; doi:10.1371/journal.pbio.3002870)
Supplement: S1 File — (DOCX) [file pbio.3002870.s001.docx]

**S1. Search strategy**

We obtaineda list of all journals indexed in MEDLINE together with their NLM ID. A random list of 400 journals was generated.

**Two potential strategies can be used to retrieve the articles:**

1. We will search for all articles published in each journal using the search strategy *1234567.jc.* where “1234567” is the NLM ID of the journal. Where NLM ID is not available, we will use the syntax “Name of journal”.nj.

We will run search for each journal separately. After each search, we will sort the results by Entry date (descending) and export the first 20 results.

Pros: All journals are equally represented.

Cons: Time consuming, as the results need to be exported manually for each journal

1. We will carry out a combined search for all journals using the following search strategy:
2. 1234567.jc.
3. 2345678.jc.
4. 3456789.jc.
5. or/1-3
6. limit 4 to dt=yyyymmdd-yyyymmdd

Pros: Faster, potentially represent the distribution of articles among journals

Cons: Unequal number of articles per journal; may end up with >20,000 articles (some journals have more than others within the same time frame), we can tweak the date range to get as close to 20,000 as possible.

**Three potential strategies can be used to obtain the email addresses of authors:**

1. All retrieved articles will be re-imported to EndNote/Zotero/Mendeley to retrieve PMID numbers. The list of PMID numbers will be exported as an .csv file and input into an R script (built based on the easyPubMed package) to retrieve the authors’ name, affiliation institutions and email addresses.

Pros: Quick & reliable results, retrieve multiple email addresses

Cons: Not working if the PubMed page does not display email addresses (usually for newly-indexed studies – we can tweak the date rate to mitigate this)

1. In addition, we will use the Find Full Text function in EndNote to retrieve PDF files of these articles, and run these files in another R script for text recognition to extract email addresses.

Pros: High rates of success

Cons: Only retrieve one email address, only work if PDF is present, sometimes false positives (e.g. publisher email address)

1. Any articles where email addresses cannot be retrieved from both methods will be manually screened.

Results from all three methods will be combined into the final list and counter-checked by another author for potential errors before survey distribution.
