# Supplementary material for: Biomedical researchers’ perspectives on the reproducibility of research
Source: PLoS Biol. 2024 Nov 5;22(11):e3002870. doi: 10.1371/journal.pbio.3002870 (PMC11537370; doi:10.1371/journal.pbio.3002870)
Supplement: S2 File — The survey was administered online and used survey logic to present relevant items. (DOCX) [file pbio.3002870.s002.docx]

**S2. Study survey**

**Demographics**

1. What describes you best?

Graduate student

Postdoctoral fellow

Faculty member/PI

Research support staff (E.g., research manager, research associate, technician)

Scientist in industry

Scientist in third sector (E.g., NGO, non-profit)

Government scientist

Other, please specify

1. What is your gender?
   1. Female
   2. Male
   3. Non-binary
   4. Prefer to self-describe:
   5. Prefer not to say

1. What country are you currently employed in? (drop down list)

1. Which of the following best describes your research area?
   1. Clinical research
   2. Preclinical research – in vivo
   3. Preclinical research – in vitro
   4. Health systems research
   5. Methods research
   6. Other, please specify

**Reproducibility perceptions**

For the purposes of this survey, we consider a study to be reproduced when its findings are confirmed in similar experimental systems (these may include slight variations in methods or materials.) By contrast, a study is replicated when it is repeated exactly. This survey talks about the larger issue of reproducibility of results, not just replication.

1. In your view, is there a reproducibility crisis in biomedicine?
   1. Yes, significant crisis
   2. Yes, a slight crisis
   3. No, there is no crisis
   4. Don’t know

1. What proportion of papers in biomedicine do you think are reproducible?

0% - 100%, in 10% increments.

Biomedicine overall

Clinical biomedical research

In vivo biomedical research

In vitro biomedical research

1. In your view, which of the factors below contribute to irreproducible biomedical research results?

Always contributes; Usually contributes; Sometimes contributes; does not contribute; unsure.

- 1. Selective reporting of the published literature
  2. Pressure to publish
  3. Low statistical power
  4. Poor statistical analysis
  5. Not enough internal replication (E.g., by the original lab/authors)
  6. Insufficient study oversight
  7. Lack of training in reproducibility
  8. Failure to make materials openly available
  9. Failure to make original study data openly available
  10. Poor study design
  11. Fraud
  12. Poor quality peer review
  13. Problems in the design of replication studies
  14. Technical expertise required for replication
  15. Variability of standard reagents
  16. Bad luck
  17. Other, please specify

**Reproducibility experiences**

1. Have you ever tried to replicate a published study YOU previously conducted and failed (i.e., re-ran an experiment but got different results from the original study)?
   1. Yes
   2. No- all replications I have completed of my own research have been successful
   3. No – I have never tried to replicate my own research

If a or b;

1. Did you publish your replication study results? Note: if you have conducted more than one replication study of your own work please respond based on your most recent study.
   1. Yes – but it took longer to publish than other papers you’ve published that were not replications
   2. Yes – and it took about the same amount of time to publish as other papers you’ve published that were not replications
   3. Yes – but it was quicker to publish than other papers you’ve published that were not replications
   4. No – I have submitted but not yet had the work accepted
   5. No – I have not yet submitted, but intend to do so
   6. No – I don’t intend to attempt to publish this study
   7. No- Journals don’t appear interested in publishing replications
   8. Other, please specify

1. What was your motivation for replicating your own study?

1. Have you ever tried to replicate a published study conducted by another team of authors and failed?
   1. Yes
   2. No- all replications I have completed have been successful
   3. No – some [< 100%] of the replications have been successful
   4. No – I have never tried to replicate someone else’s published research

If a or b;

A. Did you publish your replication study results? Note: if you have conducted more than one replication study of another groups research, please respond based on your most recent study.

- 1. Yes – but it took longer to publish than other papers you’ve published that were not replications
  2. Yes – and it took about the same amount of time to publish as other papers you’ve published that were not replications
  3. Yes – but it was quicker to publish than other papers you’ve published that were not replications
  4. No – I have submitted but not yet had the work accepted
  5. No – I have not yet submitted, but intend to do so
  6. No – I don’t intend to attempt to publish this study
  7. Other, please specify

1. What was your motivation for replicating another researcher’s study?

1. Have you ever been contacted by another researcher who was unable to reproduce a finding you published?
   1. Yes
   2. No
   3. Unsure

**Reproducibility support**

1. Does your research institution have established procedures to enhance reproducibility of biomedical research?
   1. Yes
   2. No
   3. Unsure

If yes, please describe what processes are in place to enhance reproducibility:

1. My institution would value me doing new biomedical studies more than me doing replication studies.
   1. True
   2. False
   3. Unsure

1. In my biomedical research setting it would be harder to find funding to conduct a replication study than it would be to find funding for a new study.
   1. True
   2. False
   3. Unsure

1. Are you aware of funders providing specific calls for conducting reproducibility related research (e.g., to reproduce studies, to conduct meta-science on reproducibility)?
2. Yes ; If so, which ones
3. No

1. Does your research institution provide training on how to enhance the reproducibility of research?
2. Yes, and I have taken it ; please specify
3. Yes, but I have not taken it
4. No.
5. Unsure

1. Do you think your views on reproducibility represent those of the average researcher in your field at your career stage?
2. Yes
3. No

1. Is there anything else about reproducibility you would like to share with us?

(open text)
