# Supplementary material for: Biomedical researchers’ perspectives on the reproducibility of research
Source: PLoS Biol. 2024 Nov 5;22(11):e3002870. doi: 10.1371/journal.pbio.3002870 (PMC11537370; doi:10.1371/journal.pbio.3002870)
Supplement: S3 File — In addition, we present participant perceptions of reproducibility in different research areas. (DOCX) [file pbio.3002870.s003.docx]

**S3. Supplementary Tables**

**Table 1.** Comparison of responses (in percentage) to the item asking about a reproducibility crisis between the original Nature paper and our findings, presented overall and by discipline.

|  | **Nature study data** | | | | **Current study data** | | | | | | | | | | | | | |
| --- | --- | --- | --- | --- | --- | --- | --- | --- | --- | --- | --- | --- | --- | --- | --- | --- | --- | --- |
| **Responses** | **(N= 1576, all responses)** | | **(N=203, Medicine responses)** | | **All responses (N=1626)** | | **Clinical**  **(N=816)** | | **Preclinical- in vivo (N=190)** | | **Preclinical-**  **in vitro (N=163)** | | **Health systems**  **(N=147)** | | **Methods research**  **(N=81)** | | **Other (N=227)** | |
|  | **N** | **%** | **N** | **%** | **N** | **%** | **N** | **%** | **N** | **%** | **N** | **%** | **N** | **%** | **N** | **%** | **N** | **%** |
| Yes, a significant crisis | 819 | 52 | 121 | 60 | 438 | 27 | 221 | 27 | 55 | 29 | 47 | 29 | 33 | 22 | 22 | 27 | 60 | 26 |
| Yes, a slight crisis | 593 | 38 | 59 | 29 | 730 | 45 | 344 | 42 | 96 | 50 | 83 | 51 | 73 | 50 | 47 | 58 | 85 | 37 |
| No, there is no crisis | 47 | 3 | 8 | 4 | 237 | 15 | 124 | 15 | 25 | 13 | 21 | 13 | 14 | 10 | 7 | 9 | 46 | 20 |
| Don’t know | 117 | 7 | 15 | 7 | 221 | 14 | 127 | 16 | 14 | 7 | 12 | 7 | 27 | 18 | 5 | 6 | 36 | 16 |

**Table 2.** Participant perceptions of reproducibility

| **Item** | **Response options** | **N** | **%** |
| --- | --- | --- | --- |
| What proportion of papers in biomedicine overall do you think are reproducible? | I don’t know | 264 | 17 |
|  | 0-20% | 97 | 6 |
|  | 21-40% | 268 | 17 |
|  | 41-60% | 491 | 31 |
|  | 61-80% | 394 | 25 |
|  | 81-100% | 77 | 5 |
|  | *Missing data* | 39 | - |
| What proportion of papers in clinical biomedical research do you think are reproducible? | I don’t know | 227 | 14 |
|  | 0-20% | 119 | 8 |
|  | 21-40% | 313 | 20 |
|  | 41-60% | 453 | 28 |
|  | 61-80% | 366 | 23 |
|  | 81-100% | 115 | 7 |
|  | *Missing data* | 37 | - |
| What proportion of papers in in-vivo biomedical research do you think are reproducible? | I don’t know | 364 | 23 |
|  | 0-20% | 125 | 8 |
|  | 21-40% | 303 | 19 |
|  | 41-60% | 361 | 23 |
|  | 61-80% | 334 | 21 |
|  | 81-100% | 99 | 6 |
|  | *Missing data* | 44 | - |
| What proportion of papers in in-vitro biomedical research do you think are reproducible? | I don’t know | 385 | 24 |
|  | 0-20% | 123 | 8 |
|  | 21-40% | 253 | 16 |
|  | 41-60% | 293 | 19 |
|  | 61-80% | 359 | 23 |
|  | 81-100% | 174 | 11 |
|  | *Missing data* | 43 | - |
