# Supplementary material for: Biomedical researchers’ perspectives on the reproducibility of research
Source: PLoS Biol. 2024 Nov 5;22(11):e3002870. doi: 10.1371/journal.pbio.3002870 (PMC11537370; doi:10.1371/journal.pbio.3002870)
Supplement: S4 File — (DOCX) [file pbio.3002870.s004.docx]

**S4. Additional analyses:** of survey responses to reproducibility perception items by gender. Note that we present data for males and females only; response values for other gender options were low (all n<3)

| **Item** | **Response options** | **Male**  **(N=943)** | | **Female (N=643)** | |
| --- | --- | --- | --- | --- | --- |
|  |  | **N** | **%** |  |  |
| In your view, is there a reproducibility crisis in biomedicine? | Yes, a significant crisis | 259 | 28 | 151 | 24 |
|  | Yes, a slight crisis | 437 | 46 | 283 | 44 |
|  | No, there is no crisis | 155 | 16 | 82 | 13 |
|  | Don’t know | 92 | 10 | 127 | 20 |
|  |  |  |  |  |  |
|  | *Missing data* | - | - | - | - |

Additional analyses of survey responses to reproducibility perception items by academic role

| **Role** | **In your view, is there a reproducibility crisis in biomedicine?** | **N** | **%** |
| --- | --- | --- | --- |
| Other | Yes, a significant crisis | 19 | 26 |
|  | Yes, a slight crisis | 29 | 40 |
|  | No, there is no crisis | 10 | 14 |
|  | Don’t know | 15 | 21 |
|  | Total | 73 | 100 |
| Graduate student | Yes, a significant crisis | 28 | 32 |
|  | Yes, a slight crisis | 37 | 42 |
|  | No, there is no crisis | 8 | 9 |
|  | Don’t know | 15 | 17 |
|  | Total | 88 | 100 |
| Postdoctoral fellow | Yes, a significant crisis | 31 | 24 |
|  | Yes, a slight crisis | 63 | 49 |
|  | No, there is no crisis | 13 | 10 |
|  | Don’t know | 22 | 17 |
|  | Total | 129 | 100 |
| Faculty member/PI | Yes, a significant crisis | 288 | 25 |
|  | Yes, a slight crisis | 536 | 47 |
|  | No, there is no crisis | 181 | 16 |
|  | Don’t know | 146 | 13 |
|  | Total | 1151 | 100 |
| Research support staff (E.g., research manager, research associate, technician) | Yes, a significant crisis | 16 | 30 |
|  | Yes, a slight crisis | 21 | 39 |
|  | No, there is no crisis | 5 | 9 |
|  | Don’t know | 12 | 22 |
|  | Total | 54 | 100 |
| Scientist in industry | Yes, a significant crisis | 10 | 36 |
|  | Yes, a slight crisis | 12 | 43 |
|  | No, there is no crisis | 5 | 18 |
|  | Don’t know | 1 | 4 |
|  | Total | 28 | 100 |
| Scientist in third sector (E.g., NGO, non-profit) | Yes, a significant crisis | 11 | 41 |
|  | Yes, a slight crisis | 8 | 30 |
|  | No, there is no crisis | 6 | 22 |
|  | Don’t know | 2 | 7 |
|  | Total | 27 | 100 |
| Government scientist | Yes, a significant crisis | 15 | 28 |
|  | Yes, a slight crisis | 22 | 41 |
|  | No, there is no crisis | 9 | 17 |
|  | Don’t know | 8 | 15 |
|  | Total | 54 | 100 |
