# Supplementary material for: Biomedical researchers’ perspectives on the reproducibility of research
Source: PLoS Biol. 2024 Nov 5;22(11):e3002870. doi: 10.1371/journal.pbio.3002870 (PMC11537370; doi:10.1371/journal.pbio.3002870)
Supplement: S5 File — (DOCX) [file pbio.3002870.s005.docx]

**S5.** List of training links provided by participants

| Name/Organization providing training | Link | Format of training | Open status |
| --- | --- | --- | --- |
| CITI program: Biomedical Responsible Conduct of Research, Rigor, Reproducibility and Ethical Behavior in Biomedical Research | <https://about.citiprogram.org/news/improve-study-design-to-promote-reproducibility/> | Online course | No |
| Universidade de Sao Paulo - Ribeirao Preto College of Nursing | <http://www.eerp.usp.br/research-home/> | University resources | Unclear |
| Universite de Montreal - The reproducibility of research: Issues and good practices | <https://calendrier.bib.umontreal.ca/event/3617795> | Standalone lecture | Unclear |
| Duke University School of Medicine - Reproducibility Crisis: What Can We Do? | <https://medschool.duke.edu/events/reproducibility-crisis-what-can-we-do> | University event | Unclear |
| The University of Utah | <https://education.research.utah.edu/rigor.php> | University course | No |
| UC Davis: Training Program in Molecular and Cellular Biology | <https://mcbtrainingprogram.ucdavis.edu/quantitative-approaches-molecular-and-cellular-biology-rigor-and-reproducibility> | University course | No |
| University of Oxford - Integrity and ethics training | <https://researchsupport.admin.ox.ac.uk/support/training/ethics> | University courses and workshops | No |
| Software Carpentry | [https://software-carpentry.org](https://software-carpentry.org/) | In-person and online workshops | Yes |
| Clinical Epidemiology Unit (CEU) All India Institute of Medical Sciences Ansari Nagar, New Delhi - Designing Medical Research Workshop and Thesis | <https://www.aiims.edu/aiims/events/nero_conf.htm> | In-person workshop | Unclear |
| Columbia University Irving Medical Center - Responsible Conduct of Research and Related Policy Issues | <https://www.gsas.cuimc.columbia.edu/responsible-conduct-research-and-related-policy-issues> | University Course | No |
| IECS Institute for Clinical Effectiveness and Health Policy | <https://www.iecs.org.ar/en/> | Online courses | Unclear |
| King's College London Riot Science Club | <https://www.kcl.ac.uk/events/series/riot-science-club> | Seminar series | Yes |
| Memorial Sloan Kettering Cancer Center - Responsible Conduct of Research (RCR) | <https://www.mskcc.org/sites/default/files/node/4368/documents/rtm-1008_rcr_2018updated_0.pdf> | University course | No |
| Radboud umc university medical center | <https://www.radboudumc.nl/en/research/scientific-integrity> | University courses | No |
| Brazilian Reproducibility Initiative | <https://www.reprodutibilidade.bio.br/home> | Online webinars and events | Yes |
| The University of Alabama at Birmingham Center for Clinical and Translational Science | <https://www.uab.edu/ccts/training-academy/kaizen/kaizen-r2t> | University training platform | Yes |
| University College Cork, Ireland - Epigeum Online Research Integrity Training | <https://www.ucc.ie/en/research/support/integrity/researchintegritytraining/epigeumonlineresearchintegritytraining/> | University course | No |
| Karlsruhe Institute of Technology - Theory of Science and Ethics in Biology | <https://www.botanik.kit.edu/botzell/953.php> | University course | No |
| The University of Edinburgh | <https://www.ed.ac.uk/research-office/research-talent-and-culture/research-improvement> | Standalone website resources and online workshops | Yes |
| British Neuroscience Association | [https://www.bnacredibility.org.uk](https://www.bnacredibility.org.uk/) | Standalone website resources and webinars | Yes |
| Swiss Personalized Health Network incentive | [https://sphn.ch](https://sphn.ch/) | Standalone website resources and training events | Yes |
| Society for Neuroscience | [https://www.sfn.org](https://www.sfn.org/) | Standalone website resources and virtual events | Yes |
| University of Zurich, Center for Reproducible Science | <https://www.crs.uzh.ch/en.html> | University course materials | Yes |
| Aarhus University | <https://medarbejdere.au.dk/en/administration/researchandtalent/responsible-conduct-of-research> | University course | No |
