# Supplementary material for: Biomedical researchers’ perspectives on the reproducibility of research
Source: PLoS Biol. 2024 Nov 5;22(11):e3002870. doi: 10.1371/journal.pbio.3002870 (PMC11537370; doi:10.1371/journal.pbio.3002870)
Supplement: Attachment — Submitted filename: Revision 2 Plos Bio Reproducibility Survey -2024-6-24.docx [file pbio.3002870.s006.docx]

Reviewer #1:

The meta-research article of Cobey and colleagues presents a cross-sectional survey of biomedical researchers' perspectives on the reproducibility of research. The study builds on the widely cited Nature-survey published in Nature in 2016, but provides a more biomedical-centered perspective. In total, 1630 researchers successfully participated in this study. Briefly, 72% of participants agreed that there is a reproducibility crisis in biomedicine, with the leading cause of poor reproducibility being the 'pressure to publish' (which is interesting!). Only 16% of the participants indicated that their institution had established procedures to enhance the reproducibility of biomedical research; and 83% expected it to be harder to get funding for a replication study than for a novel one. Finally, the authors emphasize the relevance of the findings for policy makers and academic leadership to create incentives and research cultures that support better reproducibility and value research quality.

Overall, this is a short, but well-written manuscript on a highly relevant topic that presents the results of a survey on (ir)reproducibility in a clear and easy-to-read way. As stated by the authors themselves, the survey directly builds on the famous Nature-survey published in summer 2016, but differs in terms of the following two points: (1) the focus is exclusively on biomedical research (the Nature survey included scientists from various different disciplines across life sciences), (2) researchers were sampled from publication lists, allowing to report a response rate (this was not the case for the Nature survey). The latter point appears to be important to reduce any bias in the participants, as not only those researchers respond to the survey that are already actively involved in reproducibility projects. Whereas I agree on the importance of the latter point, I am not convinced by the overall novelty and design of the presented work.

**We thank the reviewer for their careful reading of our submission and this concise summary. Below we have tried to address these in our revision.**

More specifically, I would like to raise the following major points:

(1)	First of all, I am not entirely convinced by the novelty of the findings or the value of the outcome that goes beyond the Nature study. The fascinating aspect of the Nature study - at least from my perspective - was that so many researchers agreed on the existence of a crisis at a time (ir)reproducibility only started to become a hot topic in science. Furthermore, it was striking that the phenomenon was reported throughout the life sciences, arguing for a general rather than a discipline-specific problem. And finally, the Nature study provided a number of interesting causes for poor reproducibility that were discussed a lot and experimentally explored over the following years (thereby already creating the incentives for change that are discussed in the present paper). The findings of the present survey more or less confirm what has been reported previously and do not go beyond the already known problems. The focus on biomedical research is interesting, but not "groundbreaking". In case of publication, I would therefore like to see more reasons for the originality of this study as well as for its expected impact on the research community.

**We aimed to provide contemporary data on researchers' perceptions of a reproducibility crisis by building off this initial Nature paper. The Nature data was collected almost a decade ago and published slightly later in 2016 -it is foreseeable that during this period changes in perceptions of reproducibility may have occurred, especially in the wake of significant science policy changes (E.g., Open science), shifts in research assessment (e.g., DORA, CoARA initiatives). In the medical space where we focus additional factors, including the COVID-19 pandemic, could too foreseeable impact reproducibility. We feel given the possible impact of time and the factors outlined above, our survey results represent an important original contemporary perspective on reproducibility. As noted, we undertook modifications to Nature’s protocol to make the results less biased; our findings provide a better source for researchers to cite to identify the contemporary situation on perceptions oof researchreproducibility. To address the reviewers point, we have now added more discussion of how our work is situated in the existing literature by stating:**

***“While our survey approach and direction of findings are consistent with the previous Nature study, ongoing monitoring of perceptions of reproducibility in the community is critical to gauge shifts over time. Indeed, conducting this same survey again in the future would allow for a temporal comparison on how perceptions and behaviors shift over time. This suggestion aligns with the approach of the EU Commission who in their report “Assessing the reproducibility of research results in EU Framework Programmes for Research******^25^” ..cont. “***

(2)	The study is purely descriptive and the authors do not present any expectations or hypotheses. Although I agree that understanding researcher perceptions is key to driving a culture change and creating training interventions, one could also argue that the Nature study already met the criteria of an explorative study, allowing for generating some more concrete hypotheses for follow-up studies. For example, one could have looked at specific hypotheses surrounding the biomedical research community. What would you expect to be specific for this research field? Is it different from other disciplines within the life sciences? If so, why?

**We have carefully discussed this feedback among the team as we anticipate other readers may have similar initial thoughts when reading the piece. To address this, we registered our protocol a priori as a descriptive study. We have not drawn on any relevant framework for which we might have grounds to make directional predictions, nor are we aware of something that would foster this. We dedicate an entire paragraph in the discussion to why we have not conducted comparison testing. We now further refine this paragraph to outline why it would be inappropriate to compare to the Nature survey results based on factors including differences in survey approach and design. We feel that setting about to conduct hypothesis testing without a clear framework would be inappropriate. The presence or absence of differences would be difficult to explain.**

**This paragraph now reads:**
***“While it may be tempting to conduct comparison testing of your results as compared to the earlier Nature replication survey, we are not positioned to do so. Our study did not aim to replicate the original Nature paper and was registered as a descriptive study. Given that we sampled exclusively biomedical researchers, and that our recruitment approach for sampling differed significantly, it would be very difficult to disentangle whether differences in results reflect sampling bias in the original study, temporal changes in the research ecosystem over time, different perceptions in biomedicine compared to research more broadly, or a combination of these factors. We know of no relevant framework that would have been appropriate to implement in order to test specific hypotheses within the biomedical community. Further, null hypothesis testing is not appropriate for determining beliefs and when hypothesis testing is used you need a formal sample size calculation***^21,22^***.***

(3)	It is not entirely clear to me, whether the survey was designed in such a way that it allows for reducing bias and identifying non-valid answers. I am not an expert in questionnaires' techniques, but I would expect to see some questions included that ask for the same thing, but with different words. From my perspective, such a validation step would be necessary to be able to extract only valid answers (and exclude those that were given simply to give any answer). The description of the survey in the Methods section appears to be rather limited in this respect. So, I would strongly recommend to provide more information about this.

**One step we took to reduce bias in self-selection was to ask participants how much they agree with the statement: “**I feel I am more concerned about reproducibility than the average researcher in my field and at my career stage”. **The survey was however purpose-built for the study drawing extensively on the Nature study and was not validated. We did not look at the Chronbach Alpha of the items on our survey as we did not seek to create a scale or to understand the shared variance, or covariance, among the distinct questions in our descriptive study. We have added the nature of the survey design to the discussion as a limitation of the work by specifying:**

***“We acknowledge several limitations in our approach. Firstly, our study survey was purpose-built, and while drawing on the Nature study, was not designed according to a particular theoretical framework. This type of approach lends itself well to the exploratory and descriptive goals of the current study; however, it makes it harder to test hypotheses or measure changes over time. A validated scale to assess perceptions of reproducibility and its cause would be of value to the community for future research in this area. Future surveys would also benefit from a more extensive approach to piloting for clarity and readability.”***

(4)	The discussion is rather short and does not go beyond the already known arguments. I therefore think that the manuscript could greatly benefit from a more in-depth analysis of the presented observations. In particular, it would be interesting to elaborate a bit more on the point that the perceived main reason was the pressure to publish. What is the expected impact of these findings and what kind of follow-up steps would be necessary to improve the situation? Is this specific for the biomedical research community?

**This is a great point, we have revised the discussion in a number of important ways to address this feedback. Firstly, we have added a new paragraph specifying the expected impacts and next steps. We have additionally contextualized the work in the scholarly landscape outside of medicine, in particular by discussing the link to the EU commission report suggested by reviewer 2. Changes are made throughout the discussion using tracked changes, a key paragraph reads:**

***“While our survey approach and direction of findings are consistent with the previous Nature study, ongoing monitoring of perceptions of reproducibility in the community is critical to gauge shifts over time. Indeed, conducting this same survey again in the future would allow for a temporal comparison on how perceptions and behaviors shift over time.*** ***This suggestion aligns with the approach of the EU Commission who in their report “Assessing the reproducibility of research results in EU Framework Programmes for Research^25^”, describe an approach to understand and monitor the progress of reproducibility over time. In the work, researchers were also asked about their perceptions of reproducibility and 60% agreed that there is a reproducibility crisis, but also overwhelmingly agreed (82%) that reproducibility is ‘important’ or ‘very important’ in their discipline. Like our findings, participants indicated several key challenges to achieve reproducibility, with issues around cultural factors being paramount. Our finding that a pressure to publish was most likely to be rated as “always contributing” to irreproducibility is consistent with the conclusions of this report. Collectively, the outcomes of this body of evidence highlight perceived causes and constraints to producing reproducible research which should be prioritized within the biomedical community and targeted with interventions and supports to create improvements over time.”***

Besides these major concerns, I do have a number of minor comments to the manuscript:

*	The definition of reproducibility given in the introduction is a rather vague one. What does "consistent with the original study" mean? Is it dependent on the p-value (i.e. is a finding significant or not?) or should the observed effect simply go in a similar direction? Furthermore, what is meant by "similar methods"? How much variation is allowed? Is reproducibility here conceptualized as "generalizability"? Please be more specific, as this is the basis for the whole manuscript.

**We have revised the intro to provide more specificity around the definitions and what is meant by “consistent with the original study”. For example, we added:**

***“This definition allows for variation in methods (e.g., conceptual and direct replications) between the original study and the reproducibility study as well as different definitions of how “consistent results” are defined (i.e., using p-value, observing results in the same direction, comparing effect sizes).”***

*	At the end of the first section of the introduction the authors use the term "replicability"? Is it defined in a different way? Or is it used as synonym for reproducibility?

**Thanks for catching this. We have revised the manuscript throughout to use reproducibility or reproducibility study in order to maintain consistency.**

*	The whole introduction is rather short, focusing on the Nature study and previous efforts to reproduce studies in different research fields. Whereas these are important aspects of the reproducibility topic, the issue is certainly much more complex and touches about various different aspects that could be mentioned here. For example, what are the discussed causes of poor reproducibility and what has been done to improve the situation? In particular, the topic of publication bias is an important one when it comes to systematic replication efforts (when trying to replicate published studies, you have to deal with the underlying publication bias that might be a reason for poor reproducibility in itself). I would therefore strongly recommend to expand the introduction a bit and include some more aspects that currently play a role in the debate.

**We now provide a discussion of a broader range of the literature on causes of poor reproducibility by referring to studies including the EU Framework Programmes for Research.**

*	The methods are only superficially reported. For example, the section about the study design should also give more information about the different phases of the study (planning, piloting, surveying). Likewise, the section about the survey should provide more information about the technique used.

How many questions were included? How was the survey organized? Were the questions presented in always the same or a randomized order? Did the authors include any kind of validation steps (see above)? Why did the authors modify some questions of the Nature study and others not?

**To address these questions, we have specified our survey approach in the methods section under the ‘survey’ header further. We now note the number of possible items on the survey and specify these were presented sequentially and using survey flow logic based on participant responses. As above we discuss now in the limitation that the survey was not validated to designed to test hypotheses rather to capture descriptive information.**

*	The survey was piloted by three researchers. Although I appreciate these piloting steps, I doubt the representativeness and generalizability of this step, as the involvement of just three scientists appears to be rather limited. Why only three? And what was changed according to the feedback of these researchers?

**The aim of this approach was to catch any overtly problematic issues with our survey questions readability or clarity. It was also done to test for issues in our implementation of survey logic I the survey flow, we have reflected this by specifying:**

***“The survey was pilot tested by three researchers (not on the team) to ensure clarity and acceptability of format and we edited the survey to address their feedback regarding clarity of survey questions and issues pertaining to illogical survey flow.”***

**We also add to the limitation as note that:**

***“Future surveys would also benefit from a more extensive approach to piloting for clarity and readability.”***

*	The assignment of codes as reported in the data analysis section is not clear to me. What exactly was done here? What are potential consequences of the codes not being blinded?

**We have specified that we used an inductive coding approach where codes are created based on the data itself, rather than upon a pre-established coding framework. We have also noted the limitation of using coders who were familiar with the study topic as a potential source of bias; however, given the descriptive nature of the work this concern is likely reduced. Examples of updates include:**

***“This inductive coding approach involves creating codes based on the data itself, rather than from a pre-established coding framework.”***

***and***

***“Another potential limitation is the use of coder for the thematic analysis who were not blind to the studies objective. While the study is descriptive in nature it remains possible that knowledge of the study aim and expertise in the reproducibility literature biased the coding approach.”***

*	At the beginning of the results section it is mentioned that the authors obtained and used listed emails from all authors on an identified paper. What does it mean exactly? Did they include more than just one author per study? If yes, this could cause kind of pseudoreplication, as the authors of one study are more dependent on each other than the authors of different studies. Publications with a huge number of authors could therefore bias the outcome towards a specific direction. Please comment on that.

**It is an empirical question as to whether authors perceptions of reproducibility vary more between papers than within. We have addressed this potential source of bias by adding the text below. We note overall that participants indicated a score of ~3, the mid-point, on our item regarding their bias towards reproducibility. This suggests that overall our sampling approach was effective.**

***“Finally, our sampling approach allowed us to obtain any listed emails on a given study article. This means that more than one of the authors of a given article in our random sample may have responded to the survey. While our random sampling approach provided a strong basis for sampling, this nuance presents the possibility for some bias as authors of different studies may vary more considerably and we have no way of knowing how survey participants were represented within or between given papers.”***

*	The results are presented on the basis of pure numbers, completed by a huge number of tables. It would be much more readable to present some simplified figures or illustrations, as it was also done in the Nature study.

**This comment is similar to that of Reviewer two. We have taken his suggestion and have now created a heat map visualization of Table 4.**

*	The reference list is not formatted consistently. It is furthermore condensed to a surprisingly low number of references that could be complemented by several additional papers (related to the point that both the introduction and the discussion could cover additional topics).

**As noted above, we have expanded the intro and discussion in order to address a broader range of the literature. A consequence of this is that the reference list has expanded. We welcome specific suggestions of relevant literature if there is something the reviewer suggests.**

*	The statements concerning the link to the Nature-study are confusing. Sometimes it is explicitly stated that the aim was not to compare the data and a few sections later it is said that this approach allowed for comparing the results to the original study. Please be more concise on the aims and the actual relation to the Nature survey.

**We agree this could have been more explicit- we have specified the relationship to the Nature paper throughout more in several spots in the manuscript. We have also refined the statement of aims by adding:**

**“While our work inspired by the 2016 Nature survey, this is not a direct replication study: we sample a different community and use a sampling approach that differs from the original study which limits the ability for direct comparison.”**

Reviewer #2:

This manuscript describes a survey investigating biomedical researchers' views about reproducibility, including perceptions of the causes of irreproducibility, experiences with reproducibility, and perceived support for reproducibility from their institutions. The study builds off the 2016 Nature survey on this topic. Below are suggestions to help clarify details of the study and to improve accessibility to a broader readership.

**Thanks for these helpful reflections, Tim. Below we have tried to address these in our revision.**

1. There appears to be missing information. I could not find S1 (search strategy), S2 (full survey), S3 (breakdown of responses), and S4 (training resources)? I don't see it in the downloaded submission file or on the OSF project linked (though I did find this file that is maybe S2: <https://osf.io/a3spd>)?

**Thanks for catching this. Our OSF folder had not been updated to be made public. All materials are there and now available openly.**

Related, the discussion refers to Table 7 as 'institutions influence on reproducibility of research' yet Table 7 is 'Thematic analysis of reasons why participants replicated their own study'.

**This was an error, and ought to have signaled towards Table 9. We have updated this to reflect the correct table and further specified the results of the relevant items. This now reads:**

***We asked several items about researchers' perceptions of their institution's support for reproducibility (Table 9) and the findings collectively suggest gaps in incentives and support to pursue reproducibility projects. For instance, with just 16% of institutions having established procedures to enhance reproducibility, and 67% of researchers perceiving their institution values novelty over replication, our results suggest that overall, researchers perceive that institutions are not doing their part to effectively support and reward research reproducibility^17^. …cont.***

Finally, the authors share information about responses to a statement of "I feel I am more concerned about reproducibility than the average researcher in my field and at my career stage", yet I don't see anything in Table 9 about this question and responses?

**Thanks for catching this. The table reference referred to the earlier paragraph and this line has been shifted upwards to be clearer. This question was reported in the text only.**

2. The paper would benefit from presenting the tables in more easily digestible ways. For example, Table 3 presented two different ways is a bit confusing. I'd recommend picking one table to present instead of both (e.g., present numbers with percentages in parathesis for any given cell like Table 4). Another example, is for Table 4 it would be helpful if it was easy to digest. Maybe a heatmap?

**Thanks for this suggestion – we now present the second version of the table alone. We have also refined the table headers to make the comparisons clearer. We also have added a visualization for Table 4 as a heat map.**

3. Have the authors looked at any correlations of responses? For example, responses for if there is a 'crisis' (slight or significant) is at 72%, yet, when asked to give their perception of what proportion are reproducible responses are at the ~50% mark. A correlation analysis to understand the relationship between these two questions might be a valuable addition. Relatedly, what do the authors make of many responses being centered around the 'sometimes contributes' category in Table 4? Did the authors look to see if any respondents gave similar responses across the board on those questions? Or was there sufficient variation?

**After discussion within the team, we have opted not to conduct these correlational analyses.** A perception of 'crisis' can vary from person to person, so a perceived percentage of reproducible research of 50%, for example, is not mutually exclusive with a 72% perceived crisis. **Conducting the correlational test would also be a post-hoc change from our protocol and may be interpreted as fishing. Finally, a correlational analysis would speak to broad relationships, but is also perhaps not the optimal way of testing for relationships.**

4. A potential limitation is the drop-off rate in the survey. It would great if the authors presented this information (graphically works well, but something to indicate where drop-off occurred). For example, there were 1630 respondents to at least one question, yet it's clear there was drop off since "A total of 724 participants responded to the item about why they replicated their own research".

**All participants completed the survey but some skipped items throughout. For example, 1630 participants took part, of which just 22 (1%) failed to respond to the item “ have you ever tried to replicate a published study you previously conducted and failed?” ; The figure N=724 refers to the number out of the denominator of folks who indicated they previously replicated their own study, (i.e., it was a sub question). We have clarified this by adding the denominator in the text as we agree this was confusing in our first draft.**

5. A limitation section would be great - for example, the 'insufficient oversight' question of causes is limited in insight (and potential bias) by the sample being so heavily faculty/PI based (i.e., those responsible for oversight).

**We have added a limitations section that outline a number of biases and relevant considerations for interpreting our findings. This section also addresses your suggestion to discuss the bias towards faculty/PI. To do so, we now note:**

***“We acknowledge several limitations in our approach. Firstly, our study survey was purpose-built, and while drawing on the Nature study, was not designed according to a particular theoretical framework. This type of approach lends itself well to the exploratory and descriptive goals of the current study; however, it makes it harder to test hypotheses or measure changes over time. A validated scale to assess perceptions of reproducibility and its cause would be of value to the community for future research in this area.*** ***Future surveys would also benefit from a more extensive approach to piloting for clarity and readability.*** ***Another potential limitation is that coders conducting the thematic analysis we’re project team members who were not blind to the study's objective. While the study is descriptive it remains possible that knowledge of the study aim and expertise in the reproducibility literature biased the coding approach.*** ***Moreover, only a small number of participants (6%) provided text-based descriptions to nuance their survey responses. It is perhaps not surprising given that the survey offered no incentive to take part, but it raises the possibility that those that took the time to respond to text-based items may differ from those who did not. Indeed, the top three most represented countries in our sample overall were the US, Canada, and the UK. While we had over 80 countries represented, our responses tended to be Western-centric which may present another bias, even in these countries tend to be among the most productive producers of research. Future research delving more specifically into recruiting from a specific country, or set of countries, could help improve our understanding of how national contexts may lead to differences in perceptions and practices related to reproducibility. Finally, our sampling approach allowed us to obtain any listed emails on a given study article. This means that more than one of the authors of a given article in our random sample may have responded to the survey. While our random sampling approach provided a strong basis for sampling, this nuance presents the possibility for some bias as authors of different studies may vary more considerably and we have no way of knowing how survey participants were represented within or between given papers.”***

6. I see in table 6, the work 'replicate' and 'replication' are used? Similarly, in the discussion the authors switch feely between reproducibility and replicability. But the authors indicated they took a broad stance in the definition of reproducibility, which I took as inclusive of words like replicate and replication? What impact might this have on the responses given? For example, is the question "Have you ever tried to replicate a published study conducted by another team of authors and failed?". Respondents might have answered this as failed to get reproducible results or failed to be able to 'repeat the study exactly' as defined in the survey? This is different than question 8 in the survey (on OSF) where the failed is defined as "re-ran an experiment but got different results from the original study). Same for asking participants about whether another researcher contacted them who was 'unable to reproduce their findings', which based on definitions shared in the survey and manuscript would give a different response than if the authors asked 'unable to replicate their findings'? The terminology is indeed complex (as the authors acknowledge in the introduction - and thus there might be influence in the responses despite the authors best attempt at preventing it).

We agree. In hindsight, we probably should have amended the original Nature study to clarify these discprencies when deploying our survey. We now acknowledge this concern in our discussion, we added:
**“An important consideration of future survey or reproducibility monitoring will be to be explicit about the language and definitions of reproducibility used – terminology in this space is complex and the same words can reflect different concepts between researchers within and between research areas. Clear definitions for reproducibility will help to ensure that differences observed in survey responses over time, or between groups, reflect more than simply differences in how surveys are interpreted. “**

7. The discussion mentions 'reproducibility networks'. I agree on the need to mention, this, but just mentioning this and providing a reference I think is insufficient - it would benefit readers who have no idea what this is to give a little more context.

We have expanded this discussion using the UKRN as an example of activities and resources. This section now reads:

***“The growth of ‘Reproducibility Networks’, national peer-led consortiums aiming to promote reproducibility and understand factors related to irreproducibility, are a promising opportunity to rectify this situation^18^. For example, the UK Reproducibility Network (UKRN) provides an opportunity for institutions to formally commit to reproducibility by joining the network, and requires a formal role within the senior management team of each member institution to support the delivery of network activities with the institution. The UKRN boasts a range of events and shared resources to support reproducibility^19^. The structure of reproducibility networks allows for harmonization but also flexibility in approach. Obtaining a sustained funding mechanism will be critical to their growth and ongoing success in terms of their long-term impact.”***

8. This EC study on reproducibility might be valuable to include in the discussion: <https://op.europa.eu/en/publication-detail/-/publication/36fa41a8-dbd5-11ec-a534-01aa75ed71a1/language-en>

**Thanks for bring this to our attention, I was not familiar with it and it is highly relevant. We have added a paragraph which contextualizes our results and the report. It reads:**

***“This suggestion aligns with the approach of the EU Commission who in their report “Assessing the reproducibility of research results in EU Framework Programmes for Research***^24^***”, describe an approach to understand and monitor the progress of reproducibility over time. In the work, researchers were also asked about their perceptions of reproducibility and 60% agreed that there is a reproducibility crisis, but also overwhelmingly agreed (82%) that reproducibility is ‘important’ or ‘very important’ in their discipline. Like our findings participants indicated several key challenges to achieve reproducibility, with issues around cultural factors being paramount. Our finding that a pressure to publish was most likely to be rated as “always contributing” to irreproducibility is consistent with the conclusions of this report. Collectively, the outcomes of this body of evidence highlight perceived causes and constraints to producing reproducible research which should be prioritized within the biomedical community and targeted with interventions and supports to create improvements over time.”***

9. Was the pilot testing with researchers include any of the authors of this manuscript? Same with the researchers who coded text response, were they any of the authors? It would be good to declare this either way.

**Yes. This was brought up by the first reviewer too and is now specified in the methods and acknowledged as a limitation (see 5 above for context).**

10. Can you share your scripts (e.g., exported files form SPSS)? It would be nice to share these for others to review and reuse.

**These are now available on OSF, we shared the data as a CSV rather than in SPSS in order to increase useability: https://osf.io/3ksvz/**

11. Can you share your anonymized data (or did you not get consent for that?)

**Consent is in place, and data is now open and available on the OSF**: https://osf.io/3ksvz/

Reviewer #3:

This was an interesting and informative survey of biomedical researchers. The methods are generally sound and the findings are interesting. My only suggestions are that the results could be presented in a more interesting and informative manner using better data visualisation (graphs, figures, colour), and that the findings could be broken down by key categories (such as country, and career stage) to gain greater insights into international variation.

**Thanks for these helpful reflections, Katherine. Below we have tried to address these in our revision.**

**Regarding visualizations, we now provide table 4 in a heat map. We also now provide visualizations of our key response item regarding whether researchers perceive a reproducibility crisis in figure 1.**

Specific points below (page numbers would have helped):

Methods:

1) Piloting by three researchers seems a bit limited - a broader range including people outside of the research group might have been more rigorous.

**Agreed. This concern was highlighted by the other reviewers too and we have now listed it as a limitation. We also specify that the members were indeed outside the group of authors. This reads:**

***“******The survey was pilot tested by three researchers (not on the team) to ensure clarity and acceptability of format and we edited the survey to address their feedback regarding clarity of survey questions and issues about illogical survey flow”….”Future surveys would also benefit from a more extensive approach to piloting for clarity and readability.”***

2) Analysis in SPSS - does this mean that the analysis code isn't available? Perhaps comment on the availability of meta-data and syntax/analysis code in the manuscript alongside data availability in the analysis section. Make it available if it currently is not.

**These are now available on OSF: https://osf.io/3ksvz/**

3)	The thematic analysis method seems a bit thin. Consider citing some of the key thematic methods papers that informed your approach (e.g., Braun and Clarke) there are slightly varying approaches.

Results:

Minor points:

1) Determinants of irreproducibility: 97 (give %). Very small number - perhaps discuss as a limitation and it would be good to know who these people were.

**This sentence now reads:
“A total of 97 (6%) participants provided a written response to elaborate on what they perceived were causes of irreproducibility.” And we added text specifically mentioning this limitation to the discussion “Moreover, only a small number of participants (6%) provided text-based descriptions to nuance their survey responses. It is perhaps not surprising given that the survey offered no incentive to take part, but it raises the possibility that those that took the time to respond to text-based items may differ from those who did not.”**

2) Provide all countries of employment in Table 1 to show how 'international' this survey is. The top few make it look very western-centric.

**This is a great point we had not considered. The survey is indeed western-centric despite our random sampling approach. Given the random sample suggesting that it may reflect global differences in publication output. We now discuss this by adding:**

**“**Indeed, the top three most represented countries in our sample overall were the US, Canada, and the UK. This means our responses tended to be Western-centric which may present another bias, even in these countries tend to be among the most productive producers of research. Future research delving more specifically into recruiting from a specific country, or set of countries, could help improve our understanding of how national contexts may lead to differences in perceptions and practices related to reproducibility.”

Major points:

1) I think the presentation of data in tables is very dry. My major suggestion for improving your paper is better data visualisation. Graphs, figures and colour would help. Think about people using your findings in presentations. These tables will look very dense on PowerPoint slides.

**As above we have taken this on board and added two distinct vizualizations that we feel better illustrate the findings for readers. We now provide table 4 in a heat map. We also provide visualizations of our key response item regarding whether researchers perceive a reproducibility crisis.**

2)	Similarly, I think there is more analysis that can be done to look at variability across nations and career stages. I would suggest nice graphs/charts in the supplementary of the key findings broken down by nationality and career stage. Here in the UK for example we've had some movement re. institutional involvement and initiatives around reproducibility e.g., institutional members of UKRN. It would be interesting to see whether that comes across in the responses around intuitional support, for example. Also the reproducibility 'movement' has been readily taken up by early career researchers with grassroots initiatives led by PhD students and post-docs gaining international reach (e.g., Science RIOT club, ReproducibiliTea). So I'd be interested to see how these responses differ by career stage. Providing more fine-grained analysis along with the analysis code will help this survey to have its greatest impact. As above, helpful for people planning to use your analysis in

presentations, discussions with their institutions ect.

Check typos in penultimate paragraph.

**Done. We have proofread the entire paper.**

Discussion:

1) Depending on what you find in the additional analysis there might be key things to pull out in the discussion.

**We provide our newly created visualizations and corresponding captions to specify findings more clearly. Our supplementary analysis provide a breakdown of responses between genders, between researchers in different biomedical research areas, and by career rank.**
