# Supplementary material for: Biomedical researchers’ perspectives on the reproducibility of research
Source: PLoS Biol. 2024 Nov 5;22(11):e3002870. doi: 10.1371/journal.pbio.3002870 (PMC11537370; doi:10.1371/journal.pbio.3002870)
Supplement: Attachment — Submitted filename: Revision Cobey et al PLOS Bio- 2024-9-11.docx [file pbio.3002870.s007.docx]

**Please see below for information on how we have addressed each of the remaining review concerns.**

a) Please truncate your Title to simply ""Biomedical researchers’ perspectives on the reproducibility of research"

**The title has been adjusted as requested.**

b) Please address the concerns raised by reviewer #2. Like him, it was my impression that the OSF deposition is incomplete, and may not be sufficient to reproduce the study.

**The data is now all made public. We share our code in a new file as per below.**

c) I note that you declare that you received no specific funding. Can you just confirm that this is correct?

**That’s correct.**

d) When you mention your ethics approval in the MS (“This study received ethics approval from the Ottawa Health Sciences Research Ethics Board”), please can you also include the protocol approval number. From the files in OSF, it looks like this is #20210856-01H...

**The REB number 20210856-01H has been added to the paper.**

d) Please address my Data Policy requests below; specifically, we need you to supply the numerical values underlying Figs 1 and 2, either as a supplementary data file or as a permanent DOI’d deposition. I note that you already have an associated OSF deposition, but the values underlying these Figs look like they may already be presented in the Supplementary Tables...? Please ensure that everything is made available.

**The numerical values are supplied on the OSF project:**

**DOI 10.17605/OSF.IO/3KSVZ ;** [**https://osf.io/3ksvz/**](https://osf.io/3ksvz/)

**They are also present in the supplementary files:**

**Table 1 has the data for Figure 1.**

**Table 2 has the data for Figure 2.**

f) Please cite the location of the data clearly in all relevant Figure legends, e.g. “The data underlying this Figure can be found in Table S1” or “The data underlying this Figure can be found in <https://osf.io/3ksvz/>"

**We have added the following line to link to the data underlying both figures to both captions, it was already on OSF:**

**“The underlying data for this figure can be found at https://osf.io/dbh2a.”**

g) Please make any custom code available, either as a supplementary file or as part of your data deposition.

**The code now compliments the data in the OSF: https://osf.io/f9u2a/**

As you address these items, please take this last chance to review your reference list to ensure that it is complete and correct. If you have cited papers that have been retracted, please include the rationale for doing so in the manuscript text, or remove these references and replace them with relevant current references. Any changes to the reference list should be mentioned in the cover letter that accompanies your revised manuscript.

**No changes are needed.**
